# Longitudinal association between sleep disturbance and inflammation, and the role of positive affect

**DOI:** 10.1111/jsr.13560

**Published:** 2022-02-08

**Authors:** Andrea Zagaria, Caterina Lombardo, Andrea Ballesio

**Affiliations:** ^1^ Department of Psychology Sapienza University of Rome Rome Italy

**Keywords:** C‐reactive protein, inflammatory markers, insomnia, mental health, positive emotion, psychopathology

## Abstract

Previous longitudinal evidence suggested that sleep disturbance (i.e., difficulties in sleep onset and sleep maintenance) may be longitudinally associated with systemic inflammation, which is involved in the pathophysiology of mental and somatic illness. The mechanisms underlying this association, however, remain largely unexplored. In the context of health psychology, a substantial body of literature showed that positive affect may have a favourable impact on immune and inflammatory response and buffer the proinflammatory effects of stress. Therefore, the aim of this study was to assess whether subjective sleep disturbance is longitudinally associated with serum high sensitivity C‐reactive protein (hs‐CRP), a marker of systemic inflammation, and whether this association is mediated by a decrease in positive affect. The data of 1894 participants aged 64.11 ± 8.02 years from the English Longitudinal Study of Ageing (ELSA) across three waves of data collection were analysed. Self‐reported sleep disturbance was assessed in 2008–2009, (wave 4), positive affect was assessed in 2010–2011 (wave 5), and hs‐CRP was assessed in 2012–2013 (wave 6). Path analysis adjusted for health‐related variables including depressive symptoms, cardiovascular disease, BMI, smoking, alcohol consume, and drug intake showed a significant direct effect of sleep disturbance to positive affect; positive affect directly predicted hs‐CRP. Lastly, an indirect effect between sleep disturbance to hs‐CRP through the mediating role of positive affect emerged. The findings suggest that sleep onset and sleep maintenance difficulties may be associated with inflammation through the mediation of low positive affect. The clinical significance of the findings should be further explored.

## 1 INTRODUCTION

Consistent evidence has shown that sleep disturbance such as difficulties falling asleep or maintaining sleep is associated with mental and somatic conditions characterised by an inflammatory nature such as cardiovascular disease (Grandner et al., 2012), inflammatory bowel disease (Ballesio et al., 2021), depressive and anxiety disorders (Hertenstein et al., 2019), and with all‐cause mortality (Lallukka et al., 2016). Meta‐analytic evidence also showed that sleep disturbance is cross‐sectionally associated with heightened inflammatory markers such as C‐reactive protein (CRP) and interleukin‐6 (IL‐6) (Irwin et al., 2016), and prospective studies suggested that sleep disturbance may even predict inflammation (e.g., Chao et al., 2015). Despite this evidence, the mechanisms underlying the association between sleep disturbance and inflammation remain poorly explored. In the context of health psychology, a substantial body of literature showed that positive affect may have a favourable impact on immune and inflammatory response and buffer the proinflammatory effects of stress (Pressman & Cohen, 2005). For instance, trait positive affect has been longitudinally associated with reduced IL‐6 (Stellar et al., 2015) and CRP (Blevins et al., 2017).

Importantly, it has also been well documented that sleep disturbance may result in a reduction of positive affect (e.g., Baglioni et al., 2010 for a review). Therefore, it is plausible to hypothesise that reduced positive affect may expose individuals with sleep disturbance to the risk of inflammation. The complex associations between sleep disturbance, positive affect, and inflammation, however, have long been neglected. Only very recently, Hunt et al. (2021) showed that young adults with higher trait positive affect produced lower IL‐6 and tumour necrosis factor (TNF) in response to two nights of experimental forced awakenings compared with those with lower positive affect. To the best of our knowledge, though, there is still no longitudinal study available examining the associations between naturally occurring sleep disturbance and inflammation, whilst testing the mediation of positive affect. To progress the field, the aim of this study was to assess whether sleep disturbance is longitudinally associated with heightened CRP, a marker of systemic inflammation, and whether this association is mediated by a decrease in positive affect in a large sample of older adults.

## 2 METHODS

### 2.1 Participants

Data were analysed from the English longitudinal study of ageing (ELSA), a prospective population‐based cohort study of older adults aged 50 years and older living in England which began in 2002 (Zaninotto & Steptoe, 2019). For the purpose of this study, three waves of data collection were considered: sleep disturbance was assessed in 2008/2009 (wave 4); self‐reported positive affect was assessed in 2010–2011 (wave 5); blood samples for neuro‐immune markers were measured in 2012/2013 (wave 6). Detailed information on the recruitment of the participants is reported in Zaninotto and Steptoe (2019).

### 2.2 Measures

#### 2.2.1 Sleep disturbance

Sleep disturbance was assessed at baseline (2008–2009, wave 4) using three items derived from the Jenkins Sleep Problems Scale (Jenkins et al., 1988). These items refer to the most frequent insomnia symptoms, which include difficulties falling asleep, frequent night‐time awakenings, and waking up in the morning feeling tired. Items were rated on a 4‐point Likert scale ranging from “not during the past month” to “three or more times a week”. Questions were referred to the previous month, and the scores were summed with higher scores indicating more sleep problems.

#### 2.2.2 Positive affect

Positive affect was assessed in 2010–2011 (wave 5) using a 13 item scale (Zaninotto & Steptoe, 2019). The participants were asked to rate how often they felt determined, enthusiastic, active, proud, interested, happy, attentive, content, inspired, hopeful, alert, calm, and excited in the previous month. Items were rated on a 5‐point Likert scale ranging from “very much” to “not at all”. A total score was computed by summing all items, with lower scores indicating higher positive affect.

#### 2.2.3 Inflammation

High sensitivity C‐reactive protein (hs‐CRP; mg/L) was assessed as a marker of systemic inflammation. hs‐CRP was assessed from blood drawn from the participant’s forearm during a nurse visit. The participants were asked to abstain from eating, smoking, drinking alcohol, or doing any vigorous exercise for the 30 min prior to the nurse visit. hs‐CRP was measured using the N Latex CRP mono immunoassay on the Behring nephelometer analyser II. Participants with CRP values >10 mg/L were excluded from the study since this could reflect current acute infection or serious illness rather than chronic inflammation. All blood samples were analysed at the Royal Victoria Infirmary laboratory in Newcastle upon Tyne, UK.

#### 2.2.4 Covariates

The analysis was controlled for a significant number of psychosocial and health‐related variables. The confounders included baseline hs‐CRP (i.e., wave 4), body mass index (BMI), depressive symptoms (i.e., CES‐D scores; Radloff, 1977) the presence of a recent diagnosis of cardiovascular diseases (i.e., high blood pressure, angina, heart attack, congestive heart failure, heart murmur, abnormal heart rhythm, diabetes, stroke diagnosis, high cholesterol), the use of any drugs prescribed by a doctor or nurse (i.e., medicines, pills, syrups, ointments, puffers, or injections), smoking, and self‐reported alcohol drinking rate in the previous 12 months.

### 2.3 Data analysis

**FIGURE 1:**
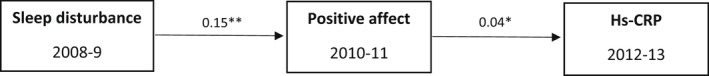
Path model. Only the simple mediation effects are graphically represented. Results are adjusted for depressive symptoms, smoking, alcohol, drugs intake, recent diagnosis of cardiovascular diseases, baseline hs‐CRP and BMI. *p < 0.05; **p < 0.001

All data were analysed using SPSS version 23 and Mplus version 8.6. Preliminarily, descriptive statistics were calculated to depict the mean and standard deviation values as well as to evaluate the distributions of the main variables. More specifically, skewness and kurtosis values greater than |1| indicated significant deviations from univariate normality (Muthén & Kaplan, 1985). Subsequently, according to the mediation model represented in Figure 1, a path analysis was employed using Mplus. Path analysis is a form of structural equation modelling (SEM) in which all variables are treated as observed variables having the advantage to study both direct and indirect effects simultaneously with multiple variables. Specifically, we adopted a focussed longitudinal mediation approach (Jose, 2016) where only the outcome was residualised. In order to test the mediation hypothesis, indirect effects tests included in the “model Indirect” Mplus software were performed. Following the recommendations of Fritz and MacKinnon (2007), we also incorporated 5000 bootstrapped samples to better estimate our mediation effect. To assess the fit of the model to the data, several fit indices were reported (Hu & Bentler, 1999): the root mean square error of approximation (RMSEA; model fit acceptable when <0.08), the comparative fit index (CFI; model fit acceptable when >0.90), the Tucker–Lewis index (TLI; model fit acceptable when >0.90), and the standardised root mean squared residual (SRMR; model fit acceptable when <0.08). The Chi‐square value was reported but not considered due to its sensibility in the case of large sample size (Hu & Bentler, 1999). Analysis was adjusted for several psychosocial and health‐related variables. Specifically, the first path (i.e., sleep disturbance to positive affect) was adjusted for concomitant depressive symptoms, while the second path (i.e., positive affect to hs‐CRP) was adjusted controlling for baseline hs‐CRP, diagnosis of cardiovascular diseases, BMI, smoking, alcohol consume, and drugs intake. The level of significance was set at *p* < 0.05.

## 3 RESULTS

### 3.1 Characteristics of the sample

Analyses were conducted on 1894 participants. Males and females were equally represented (49% and 51%, respectively). The mean age of the sample at the baseline was 64.11 (SD = 8.02). The mean body mass index (BMI) was 27.69 (SD = 4.63). A substantial percentage of the sample was married (70.3%), 5.4% cohabited, while 24.3% was single. Most of the sample (80%) did not smoke at the baseline, while only 6.7% did not have an alcoholic drink during the last 12 months.

### 3.2 Preliminary analyses

Descriptive statistics are reported in Table 1. The mean score in the sleep disturbance scale was 6.75 (SD = 2.64). The positive affect mean score reported by the sample was 29.22 (SD = 9.59). The mean score of hs‐CRP levels was 2.06 (SD = 1.86). hs‐CRP also showed a significant violation from univariate normality (both skewness and kurtosis values greater than 1). Additionally, Table 1 reports correlation analysis. The results of the latter showed that sleep disturbance significantly correlated with positive affect (*r* = 0.26, *p* < 0.01) and with hs‐CRP (*r* = 0.09, *p* < 0.01), while positive affect was significantly associated with hs‐CRP (*r* = 0.08, *p* < 0.01).

**TABLE 1:** Descriptive statistics and Pearson's product‐moment correlation coefficients between the main constructs

	M (SD)	Skewness	Kurtosis	1	2	3
1 Sleep disturbance (2008–2009)	6.75 (2.64)	0.32	−0.76	1	0.26*	0.09*
2 Positive affect (2010–2011)	29.22 (9.59)	0.51	0.16	0.26*	1	0.08*
3 Hs‐CRP (2012–2013)	2.06 (1.86)	1.67	2.68	0.09*	0.08*	1

* *p* < 0.001.

### 3.3 Path analysis

A path analysis was employed based on the path diagram represented in Figure 1. To compensate for the non‐normal distribution of hs‐CRP, model parameters were estimated using the robust maximum likelihood estimator (MLR). The model showed an excellent fit to the data: *χ* ^2^ = 18.81 (df = 8, *p* < 0.05); RMSEA = 0.027 (95% CI 0.011–0.043); CFI = 0.990; TLI = 0.979; SRMR = 0.020. The results showed a significant direct effect of sleep disturbance to positive affect (*β* = 0.15; *p* < 0.001). The positive affect also directly predicted hs‐CRP (*β* = 0.04; *p* < 0.05). Lastly, we found an indirect effect between sleep disturbance to hs‐CRP through the mediating role of positive affect (*β* = 0.006; *p* < 0.05). In order to evaluate more accurately the mediation effect, bootstrap confidence intervals were calculated using the maximum likelihood (ML) estimator (bootstrap is not available for MLR in MPlus). The 95% confidence intervals of the bias‐corrected bootstrap confirmed the statistical significance of the indirect effect (*p* < 0.05).

## 4 DISCUSSION

The present study investigated the longitudinal association between sleep disturbance and inflammation, and the mediating role of positive affect in a large sample of older adults. Consistent with meta‐analytic findings (Irwin et al., 2016), the results showed that sleep onset and sleep maintenance difficulties were associated with a higher hs‐CRP, a marker of systemic inflammation at 2 year follow up. Moreover, consistent with previous longitudinal studies (Baglioni et al., 2010 for a review), we found that sleep disturbance was associated with a reduced positive affect at 1 year follow up. Finally, we expand on prior work by showing that decreased positive affect mediates the association between sleep disturbance and inflammation. This finding is consistent with the experimental study of Hunt et al. (2021) on the role of positive affect in buffering the effects of sleep disruption on IL‐6 and TNF. More in general, our findings are consistent with the view of sleep disturbance as a risk factor for mental and physical health (Ballesio et al., 2021; Hertenstein et al., 2019; Irwin et al., 2016).

Our results are also consistent with Pressman & Cohen's hypothesis (2005), according to which positive affect may protect an individual’s physical health from the effects of stress. Several factors may explain the effects of low positive affect on inflammation. These may include behavioural (e.g., low positive affect may induce dysfunctional health behaviours), and physiological processes (e.g., low positive affect may increase sympathetic and reduce parasympathetic nervous system activity, and activate the hypothalamic–pituitary–adrenal axis) (Pressman & Cohen, 2005).

### 4.1 Limitations

The measure of sleep disturbance included in this study captures only night‐time symptoms of sleep disturbance (Jenkins et al., 1988). While this has the strength to isolate the effect of sleep onset and sleep maintenance difficulties on positive affect and inflammation, it is possible that considering daytime symptoms such as fatigue, cognitive and social difficulties which are frequently reported in individuals with sleep disturbance would have impacted our findings. Future studies are needed to advance the field by including a more comprehensive assessment of sleep variables such as the whole insomnia symptomatology, daytime excessive sleepiness, and polysomnography in order to understand the associations between specific physiological sleep features, positive affect, and inflammation. Moreover, it is also plausible to hypothesise a different direction of these associations. Since sleep and affect may be bidirectionally associated (Baglioni et al., 2010), it is possible to hypothesise that low positive affect may influence inflammation via impacting sleep. Unfortunately, ELSA data did not include the measures of interest in the time points needed to test this adjunctive hypothesis; thus, future studies in this direction are also warranted to examine a complete longitudinal mediation model in which all the autoregressive effects of the variables were addressed and consequently allowed all possible combinations of mediation to be tested. It must be also noted that we found a significant yet very small mediation effect of positive affect that may be influenced by overpowered statistical test. As *p* values should be considered in the context of sample size and clinical significance, we recommend the implementation of experimental studies (Hunt et al., 2021) to better understand the clinical significance of such associations. Finally, our study was based on adults aged 50 or older, and this is a limitation for the generalisability of the findings. Therefore, replication studies in younger populations are needed.

## CONFLICT OF INTEREST

Authors have no conflict of interest to disclose.

## AUTHOR CONTRIBUTIONS

Andrea Ballesio developed the study hypothesis and drafted the manuscript. Andrea Zagaria performed the statistical analysis and contributed to the draft of the manuscript. Caterina Lombardo revised the final draft.

## Data Availability

ELSA data are available in the UK Data Service.
